# *In vitro* irradiation system for radiobiological experiments

**DOI:** 10.1186/1748-717X-8-257

**Published:** 2013-11-01

**Authors:** Anna Tesei, Anna Sarnelli, Chiara Arienti, Enrico Menghi, Laura Medri, Elisa Gabucci, Sara Pignatta, Mirella Falconi, Rosella Silvestrini, Wainer Zoli, Vincenzo D’Errico, Antonino Romeo, Elisabetta Parisi, Rolando Polico

**Affiliations:** 1 Biosciences Laboratory, Istituto Scientifico Romagnolo per lo Studio e la Cura dei Tumori (IRST) IRCCS, Biosciences Laboratory, via P. Maroncelli 40, 47014, Meldola, FC, Italy; 2 Medical Physics Unit, Istituto Scientifico Romagnolo per lo Studio e la Cura dei Tumori (IRST) IRCCS, Meldola, FC, Italy; 3 Pathology Unit, Morgagni-Pierantoni Hospital, Forlì, FC, Italy; 4 Department of Human Anatomy and Physiopathology of the Locomotor Apparatus, University of Bologna (BO), Bologna, BO, Italy; 5 Radiotherapy Unit, Istituto Scientifico Romagnolo per lo Studio e la Cura dei Tumori (IRST) IRCCS, Meldola, FC, Italy

**Keywords:** 3-D cultures, Radiobiology, In vitro experiments, Cancer cell lines

## Abstract

**Background:**

Although two-dimensional (2-D) monolayer cell cultures provide important information on basic tumor biology and radiobiology, they are not representative of the complexity of three-dimensional (3-D) solid tumors. In particular, new models reproducing clinical conditions as closely as possible are needed for radiobiological studies to provide information that can be translated from bench to bedside.

**Methods:**

We developed a novel system for the irradiation, under sterile conditions, of 3-D tumor spheroids, the *in vitro* model considered as a bridge between the complex architectural organization of *in vivo* tumors and the very simple one of *in vitro* monolayer cell cultures. The system exploits the same equipment as that used for patient treatments, without the need for dedicated and highly expensive instruments. To mimic the passage of radiation beams through human tissues before they reach the target tumor mass, 96-multiwell plates containing the multicellular tumor spheroids (MCTS) are inserted into a custom-built phantom made of plexiglass, the material most similar to water, the main component of human tissue.

**Results:**

The system was used to irradiate CAEP- and A549-derived MCTS, pre-treated or not with 20 μM cisplatin, with a dose of 20 Gy delivered in one session. We also tested the same treatment schemes on monolayer CAEP and A549 cells. Our preliminary results indicated a significant increment in radiotoxicity 20 days after the end of irradiation in the CAEP spheroids pre-treated with cisplatin compared to those treated with cisplatin or irradiation alone. Conversely, the effect of the radio- chemotherapy combination in A549-derived MCTS was similar to that induced by cisplatin or irradiation alone. Finally, the 20 Gy dose did not affect cell survival in monolayer CAEP and A549 cells, whereas cisplatin or cisplatin plus radiation caused 100% cell death, regardless of the type of cell line used.

**Conclusions:**

We set up a system for the irradiation, under sterile conditions, of tumor cells grown in 3-D which allows for the use of the same dose intensities and schedules utilized in clinical practice. This irradiation system, coupled with 3-D cell cultures, has the potential to generate information that could be used to individually tailor radiotherapy.

## Background

The irradiation of any biological system generates a succession of processes that can be grouped into physical, physical-chemical and biological phases, all characterized by specific events occurring at different times. The principal task of radiobiology is to analyze the effects that may occur in the weeks, months and years after radiotherapy and propose improvements to current therapies.

In recent years, researchers have focused on molecular mechanisms triggered by irradiation which could help cells to escape death. Such studies are key to obtaining information on potential molecular targets for the setting up of novel radiosensitization approaches or for the design of mathematical algorithms suitable for tailoring radiotherapy [1]. It is also crucial for scientists working in this specific research area to carry out experiments on *in vitro* biological models that are capable of reproducing and mimicking clinical conditions.

Although human tumor cell lines grown in monolayer are mainly used for research and provide important information on basic tumor biology and radiobiology, they cannot be considered as fully representative of clinical tumors. Multicellular tumor spheroids (MCTS) have a three-dimensional architectural organization in which the tumor cells are not uniformly exposed to nutrients and oxygen, a condition that closely mimics the organization of human tumors. Indeed, the potential of three-dimensional (3-D) cell cultures for new anti-cancer therapeutic strategies is gaining recognition and is believed to improve the pre-animal and pre-clinical selection of the most promising treatment modalities [2-6].

The present work describes an *in vitro* irradiation system for radiobiological experiments that closely resembles clinical conditions and overcomes problems relating to spheroid irradiation under sterile conditions.

## Methods

### Cell line

CAEP cells, derived from a squamous carcinoma of the lung, were isolated and established in our laboratory, as previously described [7]. A549, a commercially available cell line derived from primary lung cancer, was obtained from the American Type Culture Collection (ATCC, Rockville, MD). Before seeding in the bioreactor culture vessels, cells were expanded and maintained as a monolayer at 37°C and subcultured weekly. Culture medium for the CAEP cells was composed of DMEM/HAM F12 (1:1) supplemented with fetal calf serum (10%), glutamine (2 mM) (Euroclone, Milan, Italy) and insulin (10 g/ml) (Sigma Aldrich, Milan, Italy). A549 cells were maintained in Ham's F12K (ATCC) supplemented with 10% FBS (Euroclone). The same culture media used for the monolayer cultures were used to grow the cells as MCTS.

### Three-dimensional cell culture bioreactor

A rotatory cell culture system (RCCS) (Synthecon, Houston, TX, USA) was used [8]. The rotator bases were placed inside a humidified 37°C, 5% CO_2_ incubator and connected to power supplies set up externally to the incubator. Culture vessels (50 mL) with a membrane that allows gas exchange were used. Vessels were rinsed in sterile PBS before all experiments. All procedures were performed in sterile conditions under a laminar flow hood. As no specific medium formulation was required for cell growth in the RCCS, we used the same medium as that utilized for the monolayer cell culture.

**Figure 1:**
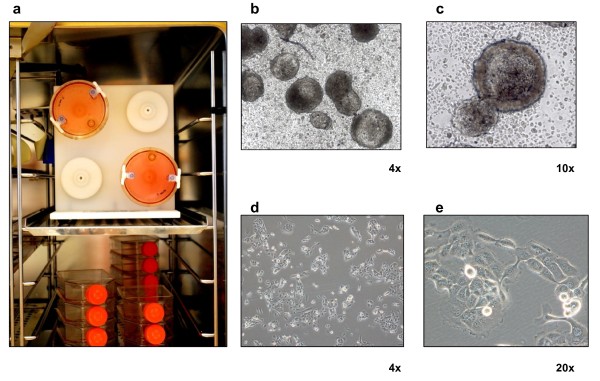
**Three-dimensional cell cultures. (a)** MCTS growing in 50-mL vessels installed into the RCCS. Representative image of CAEP grown as **(b, c)** MCTS of about 1.3 -1.5 mm ∅ sampled from about 100 spherical colonies obtained from a single vessel or grown as **(d, e)** monolayer cultures with normal bright-field or phase contrast respectively, obtained by Olympus inverted microscope with attached Nikon high speed DS-Vi1 color digital camera. Spheroid number and size were determined through image analysis using NIS-Elements D software package.

Single cell suspensions of about 1 × 10^6^ cells/ml were placed in the 50-mL rotating chamber at an initial speed of 10 rpm. As the majority of cells formed aggregates and these aggregates gradually enlarged, speed was increased over time to avoid aggregate sedimentation within the culture vessels which could hinder complete spheroid formation. The culture medium was changed every 4 days and tumor spheroids with a diameter ranging from about 700 μm to 1.3 mm (depending on the cell line used) were obtained in 15–20 days (Figure 1).

### Drug treatment

Cisplatin was freshly diluted in standard medium at a final concentration of 20 μM immediately before use. In particular, spheroids were treated by replacing 50% of the supernatant with cisplatin-supplemented standard medium using a manual 8-channel pipette. In parallel, spent medium from untreated reference spheroids was replaced with fresh drug-free standard medium.

### Irradiation system

**Figure 2:**
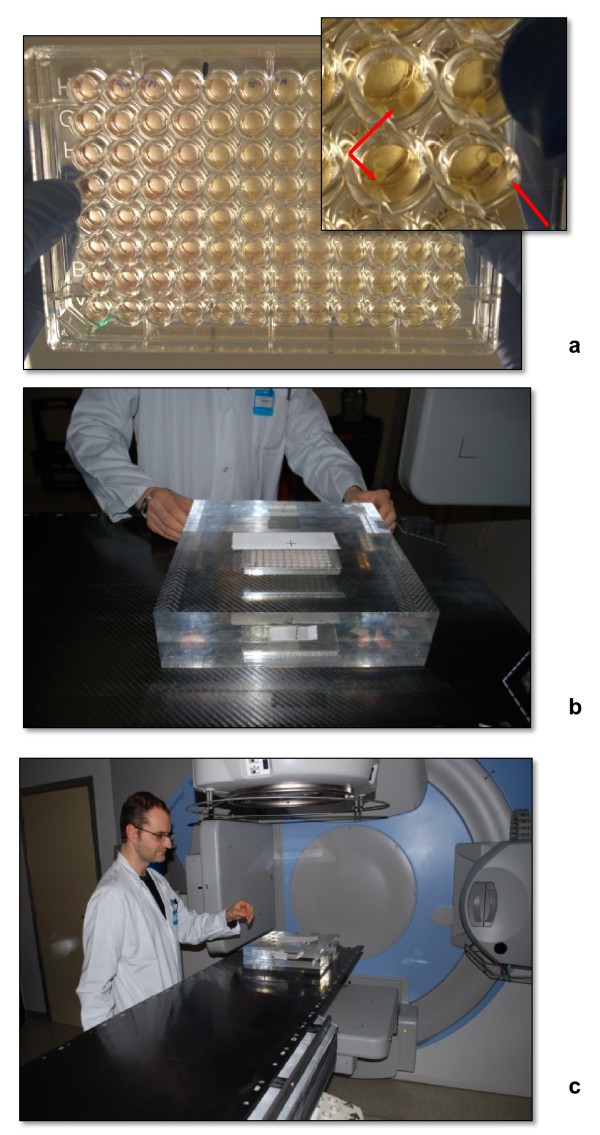
**Irradiation system. (a)** Multiwell microplate containing the MCTS ready for *in vitro* testing. The spheroids were seeded into low-attachment multiwell plates containing fresh medium alone or supplemented with 20 μM of cisplatin. The medium was removed and substituted with 250 μl of fresh culture medium after a 72-h continuous exposure and the plates were once again placed in a CO_2_ incubator at 37°C for 20 days during which time medium was changed daily. **(b, c)** Plexiglass phantom containing a microtiter plate was positioned on the treatment couch of the Elekta Synergy platform, the same machine used in daily clinical practice for the radiotherapeutic treatment of patients and periodically subject to specific quality assurance checks.

At the time of irradiation, the operator, working under the sterile laminar flow hood, transferred homogeneously-sized spheroids treated or not with cisplatin 20 μM to low-attachment 96-multiwell plates (one spheroid/well), each well previously filled with 200 μl of fresh culture medium (Figure 2a). The 96-multiwell plates containing the MCTS were inserted into a custom built plexiglass phantom (40 × 40 × 8 cm) (Figure 2b) composed of two slabs of equal size (40 × 40 × 4 cm) in which a central recess has been created at the radiation isocenter to house the microtiter plate and irradiated in a single dose of 20 Gy (delivered over about four minutes).

The same plexiglass phantom was used for the irradiation of 96-multiwell plates containing the cells grown as monolayers and exposed or not to cisplatin 20 μM.

The phantom was irradiated using a 6-MV photon beam delivered by an Elekta Synergy linear accelerator (Elekta Oncology Systems, Stockholm, Sweden) (Figure 2c), the same machine used on a daily basis to deliver radiotherapy to patients and periodically subject to specific quality assurance checks.

**Figure 3:**
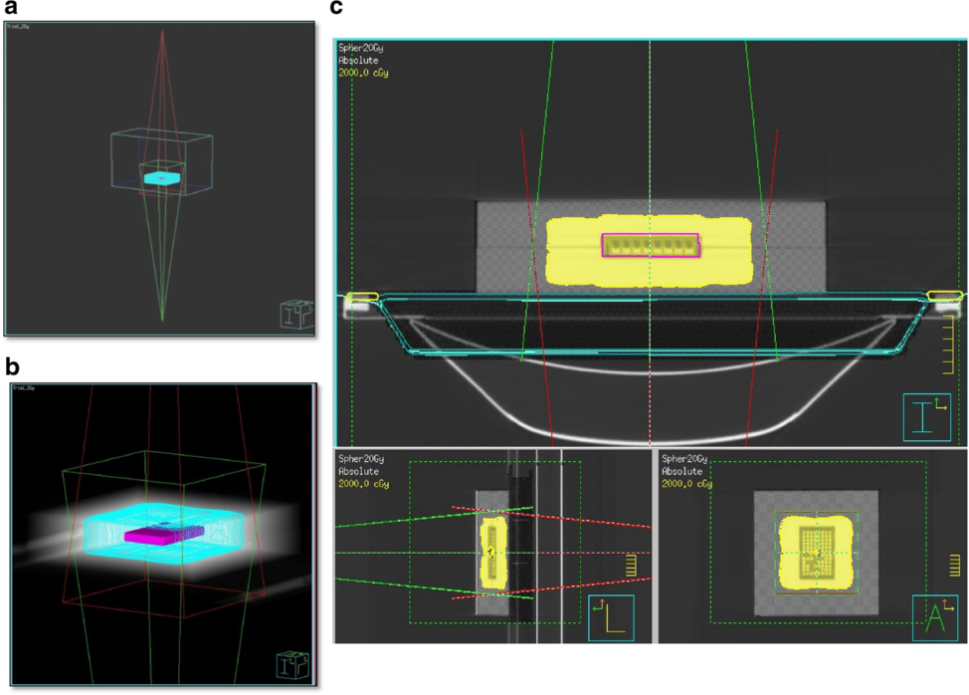
**Treatment planning for cell culture irradiation. (a, b)** Screenshot showing the source-axis distance (SAD), the dimensions of the parallel opposed fields and the target coverage used for MCTS irradiation. The SAD measured 100 cm, while the source surface distance (SSD) was 96 cm. **(c)** A CT scan was performed in the set-up phase of the phantom containing the microplate (in which wells were filled with culture medium) in order to optimize dose delivery, exactly as would be done for a patient. Two opposed fields (AP at 0° and PA at 180°) of 20x20 cm^2^ were chosen for irradiation. The irradiation system, independently of dose, ensured the delivery of a uniform dose to the yellow areas containing the microplate. A Philips Pinnacle3 radiation therapy planning system (Philips Healthcare) was used and customized with the geometric and dosimetric characteristics of an Elekta Synergy linear accelerator.

Briefly, two photon beams (parallel opposed fields of 20×20 cm^2^ defined at the machine isocenter of 100 cm from the source) were used to deliver a single dose of 20 Gy (Figure 3a,b) at the isocenter located at the centre of the recess containing the microtiter plate or the flasks (Figure 3c). The delivered dose was calculated using the Philips Pinnacle 3 radiation therapy planning system (Philips Healthcare, DA Best, The Netherlands) customized with the geometric and dosimetric characteristics of an Elekta Synergy linear accelerator. In particular, the dose distribution was calculated on a CT data-set of the water-equivalent phantom previously scanned on a Philips Brillance BigBore CT (Figure 3c). The resulting treatment plan showed an excellent calculated dose uniformity in the plexiglass phantom irradiated with two opposing beams, and the 6-MV photon energy ensured perfect equivalence between plexiglass and water (the main component of human soft tissue), as shown by the dosimetric evaluation.

Calibrated radiochromic EBT3+ films (Gafchromic EBT Films, International Specialty Products, Wayne, NJ, USA) were used to verify the exactness of dose deposition calculated by treatment planning system (TPS) Pinnacle 3. The radiochromic films were cut and placed above (surface source distance [SSD] 99 cm) and below the plate (SSD 101 cm) to record the planar dose deposition. The two pieces of film were irradiated with an open beam of 20 × 20 cm in anteroposterior (AP; film at SSD 101 cm) and posteroanterior (PA; film at SSD 99 cm) configuration and scanned after 16 h using an EPSON Expression 10000XL flat bed scanner (Epson, Milan, Italy). The net optical density (netOD) was converted to dose with home-made MATLAB software (DoseTool5) using the formalism described by Devic *et al.*[9]. The comparison performed with the open source DoseLab 4.00 (http://doselab.sf.net) between the TPS calculated dose and the radiochromic measured dose showed good, uniform agreement resulting in a gamma distribution image (Dose/Distance criteria of 5%/5 mm) with 98.4% (at SSD 99 cm) and 97.8% (at SSD 101 cm) of pixels passing the gamma test.

**Figure 4:**
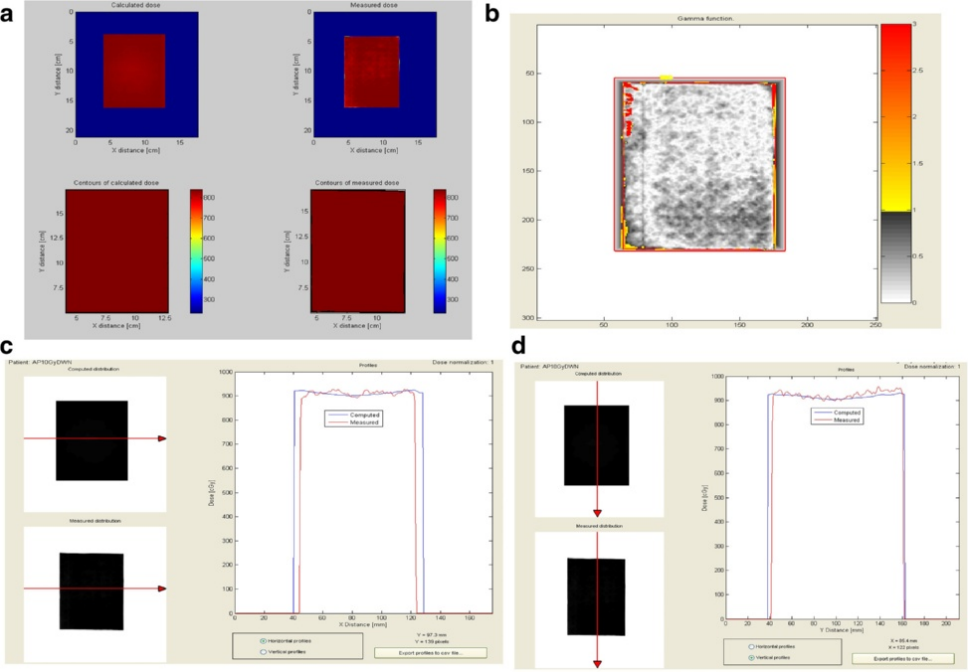
**Dosimetric evaluation of calculated and measured dose distributions. (a)** Comparison between the calculated and measured dose inside the recess holding the microplate at SSD 99 cm, evaluated using radiochromic films (Gafchromic EBT Films, International Specialty Products). **(b)** Planar distribution of gamma index representing the 98.4 pixels passing the test with dose tolerance of 5%. **(c, d)** Comparison between measured (red line) and calculated (blue line) dose profiles along the short and the long side, respectively, of the cut Gafchromic film. All images were created by Open Source DoseLab 4.00 software (http://doselab.sf.net).

A comparison between the calculated and measured planar doses at SSD 99 cm is shown in Figure 4.

**Figure 5:**
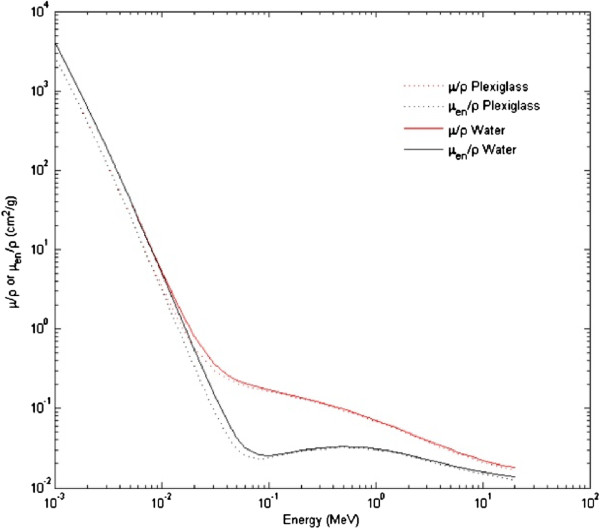
**Calculation of linear attenuation coefficient of water and plexiglass.** Comparison between the mass attenuation coefficient (red line) and the mass energy-absorption coefficient (black line) for plexiglass (dotted line) and water (solid line) as a function of the photon energy; μ/ρ = mass attenuation coefficient (for plexiglass or water), μ_en_/ρ = mass energy-absorption coefficient (for plexiglass or water); the data used for the construction of the graph are available online at http://www.nist.gov/pml/data/xraycoef/index.cfm.

A comparison between the mass attenuation coefficient (red line) and the mass energy-absorption coefficient (black line) for plexiglass (dotted line) and water (solid line) as a function of photon energy is illustrated in Figure 5. The plots for the two materials are superimposable in the range of energy of interest.

At the end of irradiation, the multiwell plates containing irradiated or cisplatin-treated and irradiated (2 Gy) spheroids were placed in a CO_2_ incubator at 37°C for 20 days during which time medium was changed daily.

### Histological staining and immunohistochemistry

Before hematoxylin and eosin staining, the cells, grown as monolayers on a Lab-Tek® Chamber Slide™ System (Nalge Nunc International, Rochester, NY, USA), were directly fixed on the slide with 10% buffered formalin. Histological staining was also performed on serial sections obtained from tumor spheres, included in collagen (StemCell Technologies), fixed in 10% buffered formalin and embedded in paraffin. Five-micrometer-thick sections were stained with hematoxylin and eosin for (morphological) photomicroscope (Zeiss, Axioscope 40) evaluation.

For the immunohistochemical determinations, anti-cleaved caspase 3 (diluted 1:200) and anti-HMGB-1 (diluted 1:400) antibodies (Cell Signaling Technology Inc., Celbio, Milan, Italy) were used. Tissue sections were treated with epitope retrieval solution [0.01 mol/L citrate buffer (pH 6.0)] in a water bath at 98.5°C for 40 min followed by a 20-min cooling period at room temperature. An anti-rabbit secondary antibody (Bond Polymer Refine, Leica Biosystems, Milan, Italy) was used to detect the primary antibodies. Cells were analyzed with a Zeiss Imager M1 microscope (Milan, Italy) and the images were recorded using a Zeiss AxioVision camera (Milan, Italy).

### Transmission electron microscopy

CAEP cells grown as monolayers or as MCTS were fixed in 2.5% glutaraldehyde in 0.1 M phosphate buffer for 2 h at 4°C and post-fixed in 1% OsO4 in 0.1 M phosphate buffer for 1 h at 4°C. Subsequently, samples were dehydrated in a graded series of ethanol and embedded in Epon resin (Sigma Aldrich, St. Louis, MS, USA). Ultrathin sections were counterstained with uranyl acetate and lead citrate and observed under a Philips CM10 electron microscope (FEI Company, Eindhoven, The Netherlands). Images were digitally captured by SIS Megaview III CCD camera (FEI Company, Eindhoven, The Netherlands).

### Determination of cell viability

Cell growth was evaluated by acid phosphatase (APH) assay [10], which has been shown to correlate with the viability of cells both composing the spheroids and grown as monolayers. Briefly (working under the laminar flow hood), homogeneously-sized tumor spheroids were selected after microscope observation and collected singly using a micropipette whose tip had been removed by a sterile scalpel. The MCTS were then plated at a density of 1.0 spheroid/well in ultralow-attachment 96-well flat-bottom plates (Corning Inc., Corning, NY, USA) to prevent their adhesion to the bottom of the wells. Plates containing MCTS were then immediately exposed to 20 μM of cisplatin for 72 h or to 20 Gy of radiation singly or in sequence (20 μM of cisplatin for 72 h followed by irradiation at 20 Gy) and washed twice by centrifugation (10 min at 400 g), after which the medium culture was replaced by H_2_O DEPC. Wells were supplemented with 100 μl of APH buffer solution (0.1 M sodium acetate, 0.1% TRITON X-100 and 10 mg of p-nitrophenyl phosphate freshly prepared before use) and incubated for 90 min at 37°C, after which 10 μl of NaOH were added to each well. Optical density of the samples was determined at a wavelength of 405 nm using a spectrophotometer plate reader. Eight-sixteen spheroids were analyzed for each treatment modality and experiments were repeated twice.

### Statistical analysis

Results were presented as the mean of at least 2 separate experiments. Statistical differences were evaluated by the Student’s t-test. Dose response curves were created by Excel software. Differences among values observed after the various treatments were analyzed using the Student’s t-test for unpaired observations. A P value < 0.05 was considered significant.

## Results

**Figure 6:**
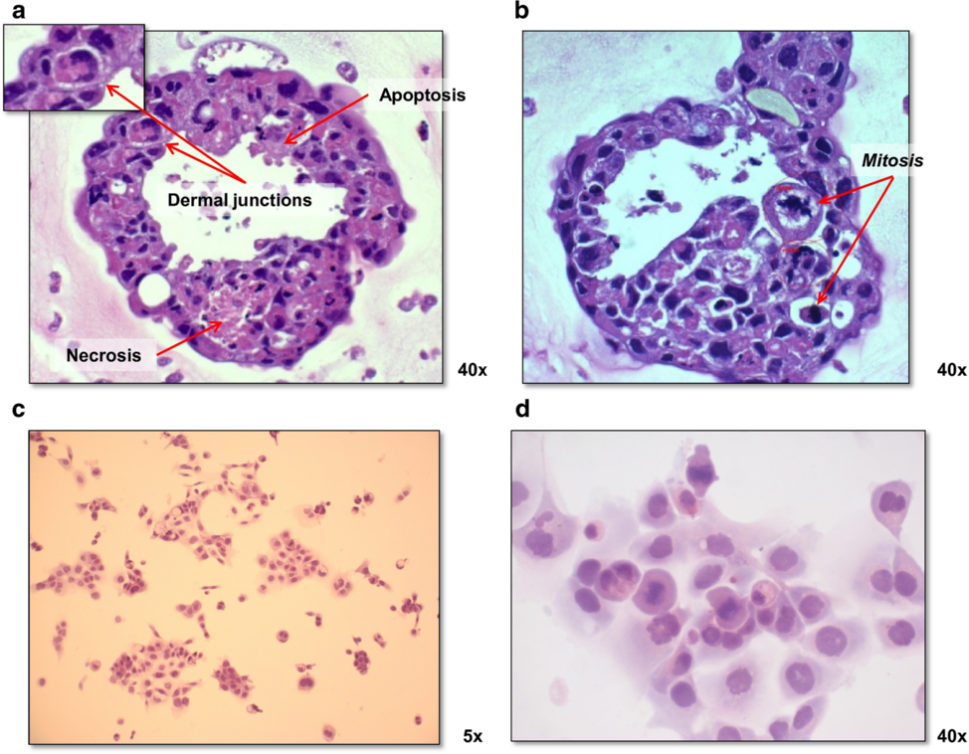
**Histological staining of cells grown as three-dimensional colonies and monolayers. (a, b)** Representative images of sections of CAEP-derived MCTS stained with hematoxylin and eosin showing pathophysiological features of solid tumors (necrosis, apoptosis, dermal junctions and mitosis). Histological staining was performed on sections obtained from tumor spheres that were included in collagen (StemCell Technologies) after 15 days of culture, fixed in 10% buffered formalin and embedded in paraffin. **(c, d)** Hematoxylin-eosin staining of CAEP cells grown on the Lab-Tek® Chamber Slide™ System (Nalge Nunc International). Before staining, cells were directly fixed on the slide with 10% buffered formalin. Photomicroscope analysis was performed using Axioscope 40 microscope (Zeiss, Milan Italy).

A bioreactor developed at NASA [8] (Figure 1a) was used to grow MCTS starting from single cell suspensions derived from established human cancer cell lines (Figure 1b-e). The pathophysiological features of solid tumors (necrosis, apoptosis, dermal junctions and mitosis) in the MCTS sections stained with hematoxylin and eosin are shown in Figure 6 a,b. In particular, areas of necrotic debris characterized by hypereosinophilia and spherical-shaped apoptotic bodies showing a loss of contact with the adjacent cells were detected. The chromatin of these cells was degraded and condensed, giving the nucleus a heterochromatic appearance. The simultaneous presence of apoptotic bodies and necrosis was confirmed by immunohistochemical analysis using specific antibodies against cleaved caspase 3 and the protein HMGB1 (Additional file 1: Figure S1), respectively. Finally, structures that could be equated with the presence of dermal junctions, normally present in tissue between adjacent cells, were observed. These features were no longer visible when the same cells were grown as monolayers. Under such conditions, numerous mitotic figures randomly distributed alongside large cells with eosinophilic cytoplasm and polymorphic nuclei were observed (Figure 6c,d).

**Figure 7:**
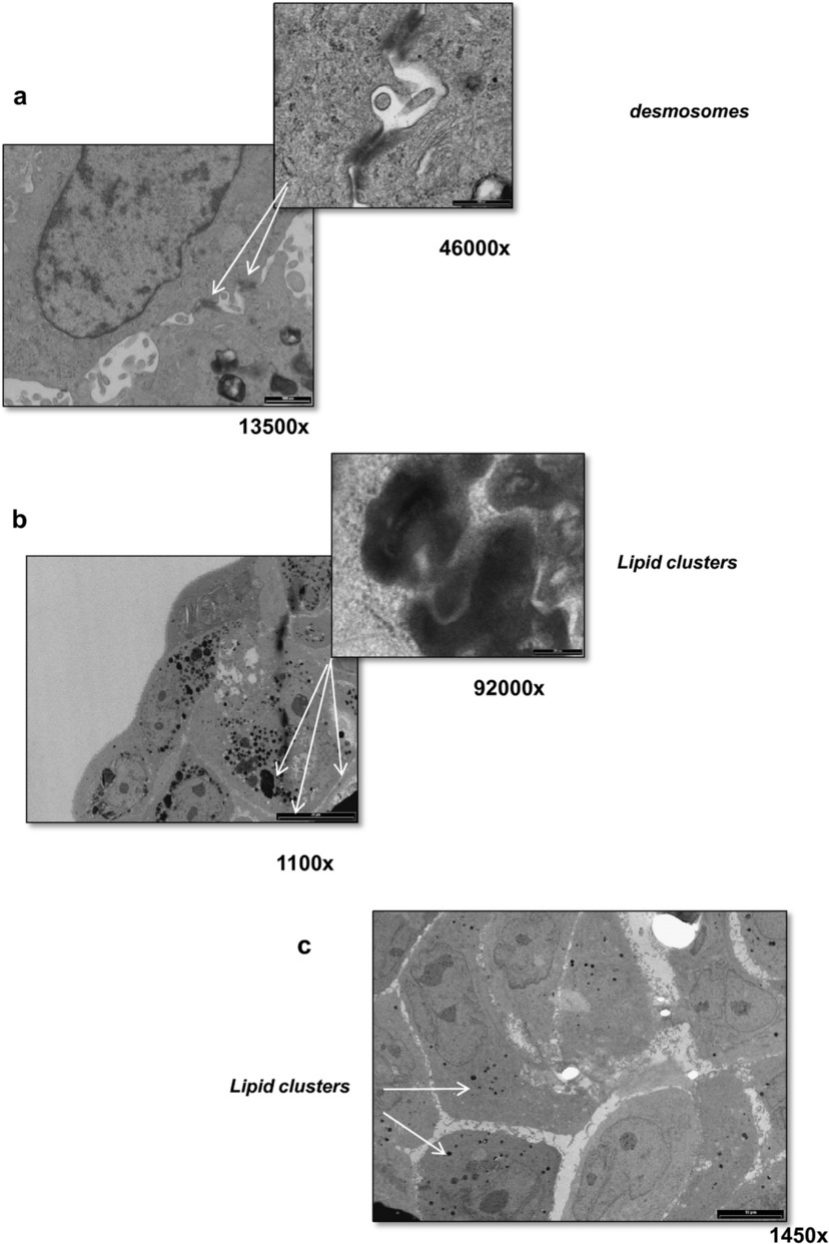
**Transmission electron microscope micrographs. (a, b)**. Representative images of 15-day-old CAEP spheroids showing features present in human lung tissue, such as desmosomes and lipid clusters. **(c)** Electron microscopy image of CAEP cells grown as monolayers. 1 × 10^4^ CAEP cells were seeded on glass slides, washed with PBS and processed as previously described in the Methods section.

In addition, ultrastructural electron microscopy analysis revealed features present in human lung tissue, such as desmosomes, confirming what hematoxylin and eosin staining had highlighted, and lipid clusters [11-13] (Figure 7a,b). Such structures were not observed in monolayer cultures of established tumor cell lines, as confirmed by the electron microscopy image of monolayer CAEP cells that showed polygonal shaped cells with no detectable desmosomes and only a few lipid clusters in the cytoplasm (Figure 7c).

**Figure 8:**
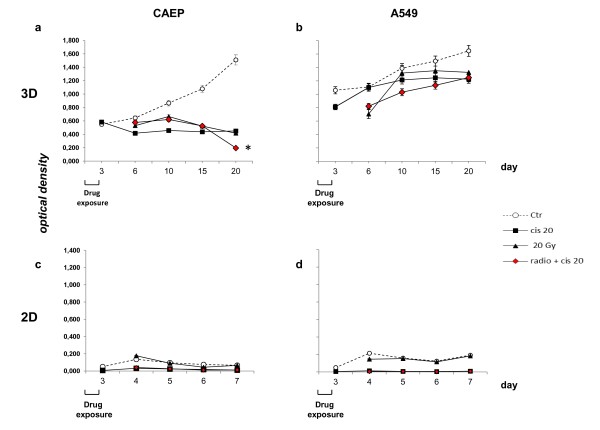
**APH assay performed on MCTS (a, b) and monolayer cell cultures (c, d) derived from human epidermoid (CAEP) and adenocarcinoma (A549) lung cancer cell lines.** The cells were treated as follows; untreated ( ; Ctr), exposed to cisplatin 20 μM (; cis 20) for 72 h, irradiated with 20 Gy (; 20 Gy) delivered in about 4 minutes, or exposed to 72-h cisplatin 20 μM followed by irradiation with 20 Gy (; radio + cis20). *P >0.05.

In the present paper we report preliminary results after a 20 Gy irradiation of CAEP- and A549–derived MCTS pretreated or not with 20 μM cisplatin (Figure 8). Results were compared with those obtained after the same treatments in cell lines grown as monolayers. The cell growth of MCTS was analyzed over a 20-day period and different effects were observed in relation to the cell line type used. In particular, in CAEP-derived MCTS, significant cell growth inhibition was observed independently of the type of treatment used (Figure 8a). Furthermore, a significant increase in radiotoxicity was observed 20 days after the end of irradiation in the spheroids pre-treated with cisplatin compared to those treated with cisplatin or irradiation alone. Conversely, although the growth of A549-derived MCTS was initially strongly hampered by the combined radio-chemotherapy treatment, at the end of the 20-day period such inhibition was similar to that induced by cisplatin or irradiation alone (Figure 8b).

We also tested the same treatment schemes on monolayer CAEP and A549 cells (Figure 8c,d), analyzing cell growth over a 7-day period. Notably, the duration of the experiment was significantly shorter than that used for 3-D cultures because the cells reached confluence about seven days after the end of treatment, showing cell-cell contact inhibition and a consequent growth arrest. Under such conditions, the different treatments tested induced the same effect independently of the type of cell line used. In particular, while 20 Gy of radiation in a single dose did not influence cell growth, both cisplatin alone and cisplatin plus 20 Gy radiation caused 100% cell death.

## Discussion

Although the use of tumor cell lines grown in monolayer provides very important information on basic tumor biology and radiobiology, it does not fully represent clinical tumors. In particular, compared to cells cultured on a flat surface, 3-D cell culture techniques better mimic the natural environments of tissues and organs, where cells exist in a 3-D microenvironment with intricate cell-cell and cell-matrix interactions and complex transport dynamics for nutrients and cells [14,15]. Furthermore, tumor spheroids show a three-dimensional organization in which cells are not uniformly exposed to oxygen or nutrients, which is more representative of the organization of human tumors consisting of regions of regularly dividing cells, hypoxic cells, and necrosis [6,16]. Conversely, monolayer cultures consist of cells generally growing in a nutrient rich liquid environment or on an agar surface with an ample supply of oxygen, which form a virtually homogeneous colony.

In the present study, MCTS were grown using a bioreactor developed at NASA [8] which exploits the low force of gravity to assemble tumor cells and, importantly, offers the advantage compared to other systems (soft agar, liquid overlay, hanging drop, etc.) of producing a large amount of homogeneously-sized spheroids. In particular, the MCTS obtained using this approach exhibit growth characteristics and pathophysiological features of avascular tumor nodules and also of peripheral tumor microregions near blood capillaries [6]. Furthermore, it must be underlined that the MCTS experimental model permits several analytical investigations in whole (growth kinetics, histology, immune cell infiltration capacity and cell mediated cytotoxicity) or dissociated spheroids (molecular analysis, re-growth analysis, clonogenic assay, flow cytometry, biochemical assay) to be performed at baseline or after radiation treatment. Several studies have shown that cancer cells grown in a 3-D culture survive low doses of chemotherapeutic agents that would otherwise be cytocidal in the same cells grown in a two-dimensional (2-D) culture [6,17]. Our preliminary results highlighted differences in terms of chemo- or radiosensitivity of the two cell lines grown as 3-D spheroidal colonies after a 20-day observation, a culture time that cannot be reached by 2-D cultures (maximum 7–8 days).

In the present work we also described how we resolved some of the problems relating to irradiation of the cells (the same system used for MCTS can be used to irradiate flasks or plates containing cell cultures grown as monolayer) under sterile conditions. For example, some authors place the cell-containing plates or flasks, wrapped in cling-film and tape, in basins filled with water to simulate the passing of radiation through tissues. These experimental conditions run the risk of water infiltration which could compromise the sterile conditions. We, on the other hand, inserted the 96-multiwell plates or the flasks containing the cells into a custom-built plexiglass phantom. Plexiglass was chosen because, from a physical point of view, in terms of interaction with radiation, it is the material most similar to water, the main component of human tissues passed through by radiation beams before reaching the target tumor mass. Furthermore, the recess within the plexiglass slabs was tailored for spheroid-containing microplates and used in our experiments to minimize the interfaces of different density between the plexiglass walls and that of the microplate itself. In this way, the plate containing the spheroids was closed inside the plexiglass chamber by its lid, thus avoiding further manipulations and the risk of water infiltration. This system also guarantees irradiation of the 3-D or 2-D cultures with complete coverage of the target utilizing the same equipment and calculation methods used for the planning of patient/treatments, without the need for dedicated or highly expensive instruments and dosimetric verification for each experiment. Furthermore, the duration of dose delivery ranges from a few seconds to a few minutes, which does not influence cell survival. We are thus able to perform experiments with both conventional fractionation and hypofractionation regimens using the same procedures and timing as routine clinical practice, resulting in a more realistic evaluation of the cellular and biomolecular effect of radiation therapy.

## Conclusion

We believe that the novel system and biological model proposed represents an important experimental approach for researchers working in the area of radiobiology that could deepen our understanding of the basic mechanisms of tumor radiobiology and help to improve clinical treatment modalities.

## Abbreviations

2-D: Two-dimensional; 3-D: Three-dimensional; MCTS: Multicellular tumor spheroids; APH: Acid phosphatase; RCCS: Rotatory cell culture system; SAD: Source-axis distance; SSD: Source surface distance.

## Competing interests

The authors declare that they have no competing interests.

## Authors’ contributions

AT designed and carried out the experiments, developed the methodology described in the manuscript, contributed to the acquisition, analysis and interpretation of data, and wrote the paper. AS, EM and CA contributed to the development of the methodology described in the manuscript and to the analysis and interpretation of data. EM, EG, SP, LM, MF, VD’E, AR and EP carried out the experiments described in the manuscript and were involved in data interpretation. RS and WZ supervised the research and reviewed the manuscript for important intellectual content. RP helped to design the experiments and to develop the methodology described in the manuscript, and contributed to data analysis/ interpretation and to the writing of the manuscript. All authors read and approved the final manuscript.

## Supplementary Material

Additional file 1: Figure S1 Immunohistochemical (IHC) detection of apoptotic and necrotic cells. (a) Representative image showing cytoplasmic and perinuclear localization of cleaved caspase 3 in apoptotic cells (low and high magnification) using a specific anti-cleaved caspase 3 antibody. (b) IHC staining for cellular necrosis using a primary anti HMGB1 protein antibody. Notably, in this case positivity was localized in the central area of the spherule, which was composed mainly of cellular debris. Both IHC stains were performed on paraffin-embedded CAEP tumor spheres using primary antibodies and detected with Bond Polymer Refine detection system (Leica Biosystems). Click here for file
