# Supplementary figures and images for: In vitro irradiation system for radiobiological experiments
Source: Radiat Oncol. 2013 Nov 1;8:257. doi: 10.1186/1748-717X-8-257 (PMC3874638; doi:10.1186/1748-717X-8-257)

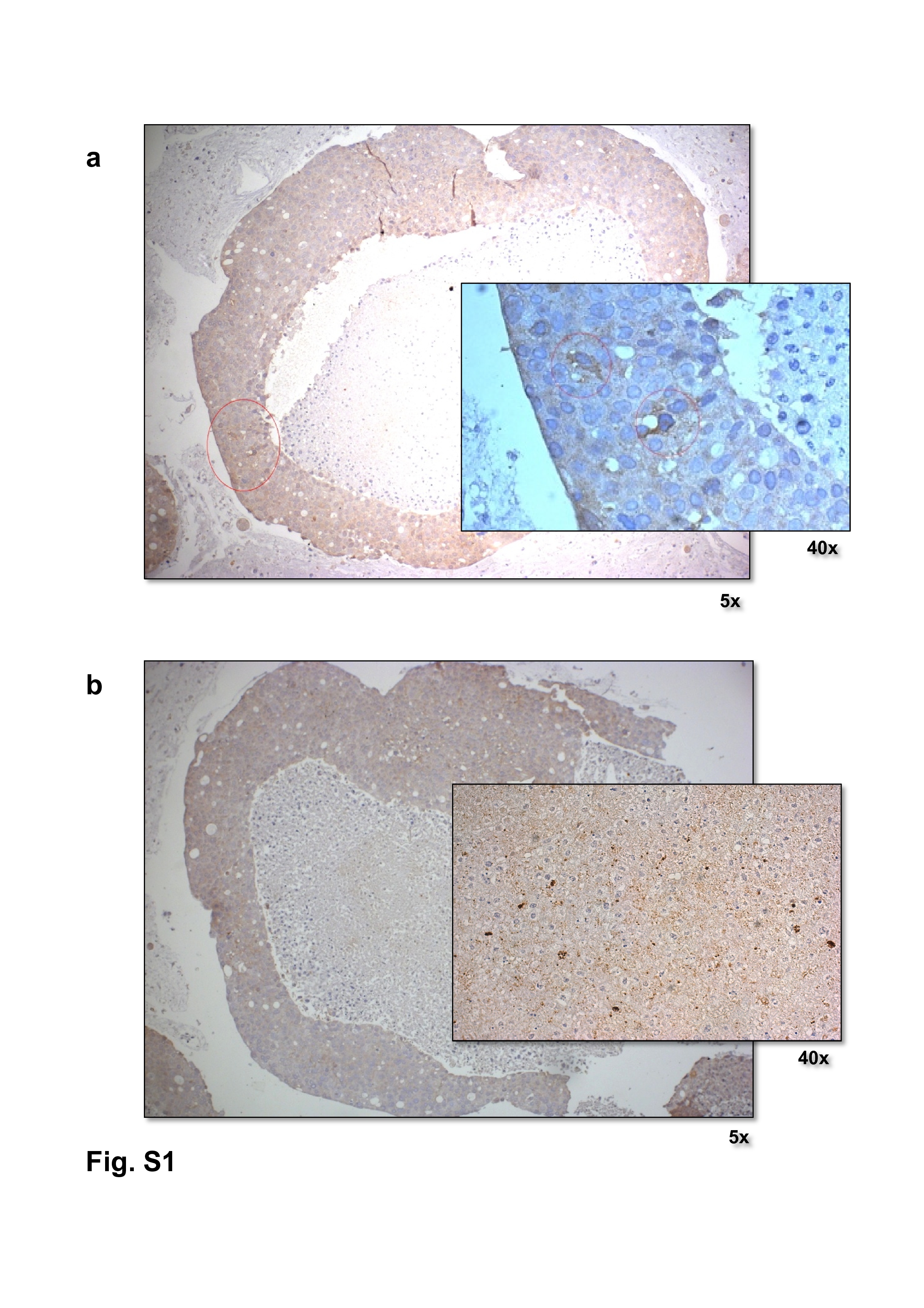

Supplement: Additional file 1: Figure S1 — Immunohistochemical (IHC) detection of apoptotic and necrotic cells. (a) Representative image showing cytoplasmic and perinuclear localization of cleaved caspase 3 in apoptotic cells (low and high magnification) using a specific anti-cleaved caspase 3 antibody. (b) IHC staining for cellular necrosis using a primary anti HMGB1 protein antibody. Notably, in this case positivity was localized in the central area of the spherule, which was composed mainly of cellular debris. Both IHC stains were performed on paraffin-embedded CAEP tumor spheres using primary antibodies and detected with Bond Polymer Refine detection system (Leica Biosystems). [file 1748-717X-8-257-S1.jpeg]
